# Human Nails Permeation of an Antifungal Candidate Hydroalcoholic Extract from the Plant *Sapindus saponaria* L. Rich in Saponins

**DOI:** 10.3390/molecules26010236

**Published:** 2021-01-05

**Authors:** Vanessa Mendes, Flávia Franco Veiga, Lidiane Vizioli de Castro-Hoshino, Francielle Sato, Mauro Luciano Baesso, Beatriz Vesco, Elton Cruz, Izabel Cristina Piloto Ferreira, Melyssa Negri, Terezinha Inez Estivalet Svidzinski

**Affiliations:** 1 Departamento de Análises Clínicas e Biomedicina, Universidade Estadual do Paraná (UEM), Avenida Colombo 5790, 87020-900 Maringá-PR, Brazil; vmplanas@gmail.com (V.M.); flaviafveiga@gmail.com (F.F.V.); beatrizvesco1@gmail.com (B.V.); me.eltoncruz@gmail.com (E.C.); mfnngrassi2@uem.br (M.N.); 2 Departamento de Física, Universidade Estadual de Maringá (UEM), Avenida Colombo 5790, 87020-900 Maringá-PR, Brazil; lidyvizioli@gmail.com (L.V.d.C.-H.); fsato@uem.br (F.S.); mlbaesso@dfi.uem.br (M.L.B.); 3 Departamento de Farmácia, Universidade Estadual de Maringá (UEM), Avenida Colombo 5790, 87020-900 Maringá-PR, Brazil; icpferreira@uem.br

**Keywords:** *Sapindus saponaria* L., *Trichophyton* spp., antifungal, human nail, permeation, onychomycosis

## Abstract

We evaluated a hydroalcoholic extract of *Sapindus saponaria* L. pericarps (ETHOSS), as a candidate to a topical antifungal medicine for onychomycosis. ETHOSS was produced by extracting the crushed fruits in ethanol. The saponin contents were identified and characterized by electrospray ionization mass spectrometry. We measured the in vitro antifungal activity against three dermatophyte fungi, isolated from onychomycosis: *Trichophyton rubrum*, *T. mentagrophytes,* and *T. interdigitale*, using broth microdilution tests. The minimum fungicide concentration of ETHOSS ranged from 195.31 to 781.25 μg/mL. The cytotoxicity of the crude extract was tested on the HeLa cell line, and its ability to permeate into healthy human nails by photoacoustic spectroscopy and Fourier transformation infrared spectrometer (FTIR) spectroscopy by attenuated total reflection. Besides its strong antifungal activity, ETHOSS showed low cytotoxicity in human cells. It was able to permeate and reach the full thickness of the nail in one hour, without the aid of facilitating vehicles, and remained there for at least 24 h. These results suggest that ETHOSS has great potential for treating onychomycosis.

## 1. Introduction

Onychomycosis (OM) is a fungal infection of the human nail, prevalent across the globe [1], with a significant negative impact on the physical, functional, psychosocial, and emotional aspects of patients’ lives. This infection is contagious and may spread to other nails and other people [2]. Even though this disease affects people of both genders and all ages, it particularly targets the elderly and immunocompromised patients [3]. The prevalence of OM in the general human population was estimated to be between 2% and 9%, with an increasing incidence reported in recent years [4,5,6].

OM can have many clinical aspects, and several oral and topical treatment options are available [7]. Oral therapies include administration of allylamines, and azoles, while for topical application, ciclopirox, amorolfine, efinaconazole, and tavaborole are used. Terbinafine is good as both an oral and topical drug [8]. Oral antifungals are often the first line of treatment, and their efficacy is high; however, due to their hepatotoxic effects, they are not recommended for high-risk patients. In any case, the present set of drugs to treat OM have a number of limitations, such as difficulty in nail permeation of topical drugs, slow nail growth, extended duration of treatment, and frequent adverse reactions [2]. Currently, there are very few oral antifungal options available in the market, particularly for vulnerable patient groups such as the elderly and those with poor hepatic health [8]. Therefore, topical therapy seems to be an advantageous alternative for the treatment of OM for these patients, as it is applied directly to its site of action, eliminates hepatic metabolism, thus minimizing adverse reactions and reducing the risks of toxicity. However, there are limited topical drugs available [9], necessitating the search for new treatments. Natural compounds, like plant extracts, would be advantageous in this context to circumvent the use of synthetic drugs.

The fruits of *Sapindus saponaria* L. (Sapindaceae), commonly found in tropical regions, are rich in saponins and glycosides, which chemical structure has a hydrophilic and a hydrophobic portion [10]. They can be a source of an interesting set of compounds for treating fungal infections, as plant-derived saponins are promising antifungal drugs [11].

Previous studies with *S. saponaria* extracts showed excellent inhibitory and fungicidal actions in vitro [10] and in vivo [12]. These results suggested this extract as a promising topical drug for treating OM. However, it has not been tested on dermatophytes, the most common group of fungi that cause OM. Their cytotoxicity in human cell lines and their ability to permeate the nail surface is also unknown.

The main agents of OM are dermatophyte fungi, from the genus *Trichophyton* [13], particularly species of *T. rubrum* and *T. mentagrophytes* complexes [9], including the anthropophilic species *T. interdigitale* [14].

Thus, the aim of this study was to evaluate the in vitro antifungal activity of the hydroalcoholic extract of *S. saponaria* (ETHOSS) on dermatophyte fungi and to verify its cytotoxicity and permeance through the human nail.

## 2. Results

### 2.1. Analysis of Phytochemical Contents of ETHOSS

**Figure 1:**
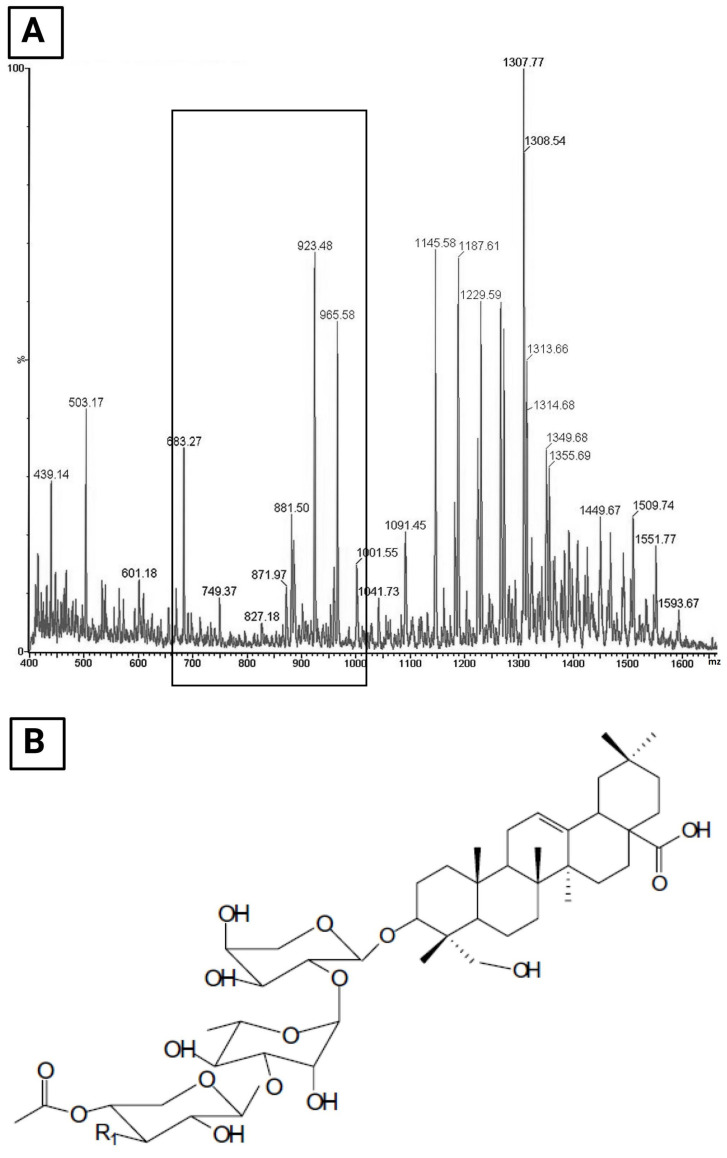
Electrospray ionization mass spectra (ESI-MS) of H and E (hematoxylin and eosin) obtained from *Sapindus saponaria* hydroalcoholic extract (ETHOSS) rich in saponins, highlighting the region between 650 and 1000 m/z (**A**). The molecular structure of a saponin (**B**).

The phytochemical analysis of ETHOSS (Figure 1) shows peaks of molecular fragments and ions between 400 and 1550 m/z (Figure 1A). Absorption peaks were seen at 683.27, 881.50, 923.48, and 965.58 m/z, are the characteristic of saponins. The molecular structure of saponin is shown in Figure 1B.

### 2.2. In Vitro Antifungal Activity of ETHOSS against Pathogens of Onychomycosis

The minimum inhibitory concentration (MIC) and minimum fungicidal concentration (MFC) of ETHOSS against *T. rubrum* CMRP 2913, *T. mentagrophytes* CMRP2920, and *T. interdigitale* CMRP 2921 are shown in Table 1. The results indicate that the hydroalcoholic fraction of the extract was effective against these dermatophytes. In addition, the minimum inhibitory concentration (MIC) and minimum fungicidal concentration (MFC) values were comparable, ranging from 195.31 μg/mL to 781.25 μg/mL.

### 2.3. In Vitro Cytotoxicity Assay

**Figure 2:**
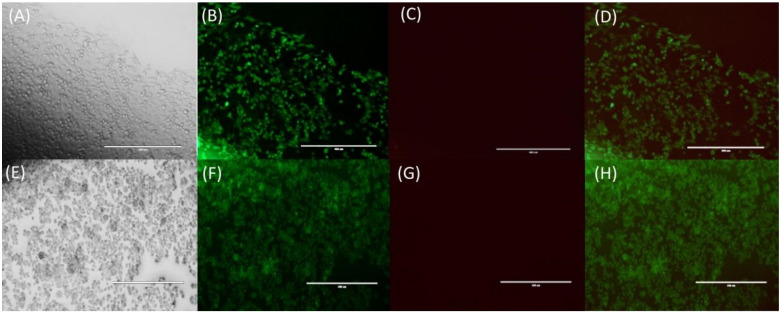
HeLa cells stained with Live/Dead^®^ kit, scale bar = 400 μm observed under an EVOS FL microscope, Life Technologies, Carlsbad, CA, USA. The assay shows live cells stained with calcine-AM (green) and dead cells, evidenced by ethidium-1 homodimer (red). (**A**–**D**) Designate the normal morphology of the untreated cell line. (**E**–**H**) Represent cells after treatment with 781.25 μg/mL ETHOSS for 24 h. (**A**,**E**) Cells observed by a phase-contrast microscope. (**B**,**F**) Live cells stained with Live/Dead^®^ kit and observed by fluorescence microscope with fluorescein optical filter, 485 ± 10 nm. (**C**,**G**) Dead cells stained with Live/Dead^®^ kit and observed by fluorescence microscope with fluorescein optical filter, 530 ± 12.5 nm. (**D**) Represents the overlap of (**B**,**C**). (**H**) Represents the overlap of (**F**,**G**).

The cytotoxicity of ETHOSS to mammalian cells was assessed by determining the viability and morphology of HeLa cells by Live/Dead^®^ staining. Results show that at a concentration of 781.25 μg/mL, ETHOSS induced slight changes in the typical morphology of the HeLa cells (Figure 2). However, these cells remained viable (green color).

### 2.4. Ex-Vivo Study on the Permeance of ETHOSS into Human Nails

**Figure 3:**
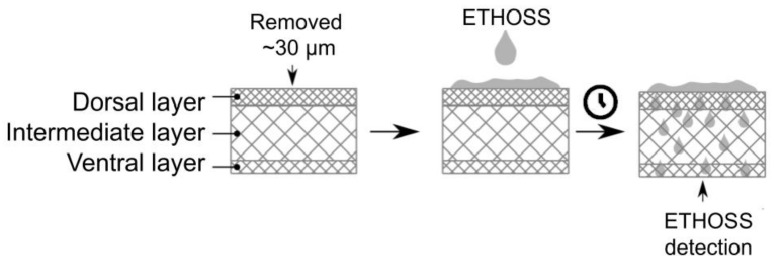
Scheme adapted from Veiga et al. [15], illustrating the anatomy of the nail with its three regions: dorsal (external), intermediate, and ventral (internal). The application of ETHOSS on the dorsal side and evaluation of its permeation to the ventral side. It was also applied on the ventral surface, following a similar scheme not represented graphically.

The ability of ETHOSS to permeate the human nail was evaluated by applying it on one side of the nail surface and detecting it on the reverse side (Figure 3).

#### 2.4.1. Spectrometric Characterization of the ETHOSS

**Figure 4:**
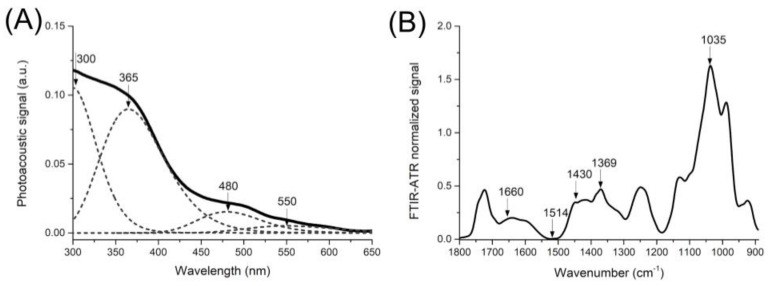
Spectrophotometric characterization of the crude hydroalcoholic extract obtained from *Sapindus saponaria* pericarps (ETHOSS) according to Photoacoustic Spectroscopy (PAS) (**A**) and Fourier transformation infrared spectrometer (FTIR-ATR) (**B**).

To evaluate the penetration of ETHOSS into the nail, the crude extract was first characterized by spectrometric analysis. Figure 4A graphically shows the Photoacoustic Spectroscopy (PAS) measurements detected by optical absorption. A broad absorption band between 300 and 650 nm was observed. Peaks at 300, 365, 480, and 550 nm were seen after adjusting the Gaussian functions to decompose the spectrum into its constituent bands, and it showed the greatest contribution of the peak at 365 nm.

These findings were confirmed by Fourier transform infrared attenuated total reflectance (FTIR-ATR), which also revealed the spectral characteristics of saponins in ETHOSS. This technique provides structural and molecular data and identifies the presence of saponin-specific functional groups in the range of 890 to 1800 cm^−1^. These also showed prominent absorption peaks at 1660, 1514, 1430, 1369, and 1035 cm^−1^, among which the largest was at 1035 cm^−1^. Detection of these characteristic peaks, mainly at 365 nm and 1035 cm^−1^ (A and B, respectively), suggested the presence of saponins.

#### 2.4.2. Spectrometric Characterization of the Nail before Treatment with the Extract

**Figure 5:**
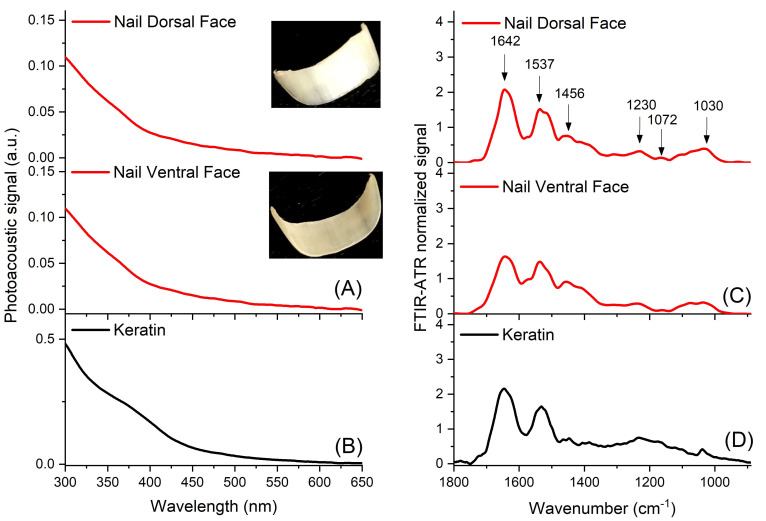
Spectra of human nail without extract (Dorsal Control and Ventral Control) and commercial keratin (Keratin) obtained separately by the PAS technique (**A**,**B**) and by FTIR-ATR (**C**,**D**), respectively.

Figure 5 shows the spectra from the dorsal and ventral surfaces of the nail prior to the application of the ETHOSS, and of the commercial keratin. The first column shows the spectra from PAS, and the second column contains the FTIR-ATR readings. The major component of the nail is keratin, as seen by comparing commercial keratin (Figure 5A–D). A broad absorption band of 300 to 650 nm (Figure 5A) and more defined peaks from 890 to 1800 cm^−1^ (Figure 5C), centered at 1642 (V (C=O): amide I), 1537 (amide II), 1456 (amide II), 1230 (amide III), 1072 (skeletal (CC) of DNA), and 1030 cm^−1^ (V (CC): keratin), according to Veiga et al. [15].

### 2.5. Nail Permeation of ETHOSS

**Figure 6:**
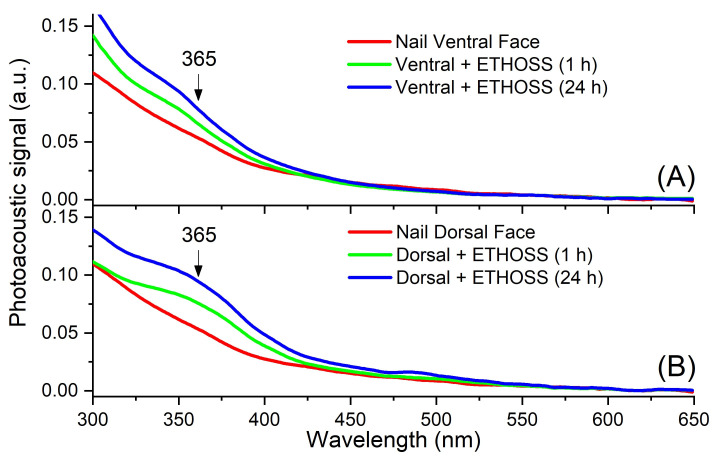
Nail PAS spectra 1 and 24 h after ETHOSS application (100 mg/mL), both on the ventral (**A**) and dorsal (**B**) faces. There was permeation of the extract through the total thickness of the nail, regardless of the face in which it was applied.

The PAS spectra were read 1 h and 24 h after the application of ETHOSS on dorsal and ventral surfaces of the nail (Figure 6A,B). In both situations, there was an increase in the intensity of absorption between 300 and 450 nm, where there was a greater concentration of ETHOSS (peak of 365 nm), as explained in Figure 4, indicating that the extract was able to permeate the entire nail after 1 h of application (green line) and remained even after 24 h (blue line).

**Figure 7:**
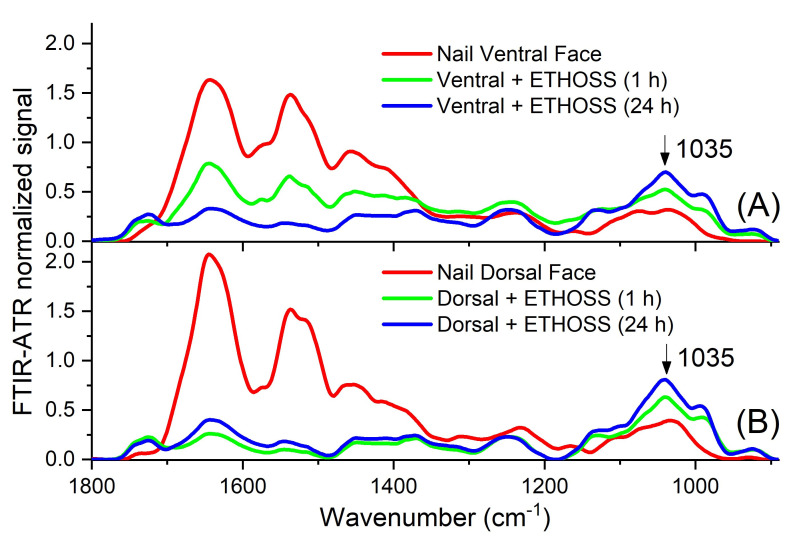
FTIR-ATR spectra of the nail after 1 and 24 h of ETHOSS application (100 mg/mL), both across the dorsal (**A**) and ventral (**B**) faces. There was permeation of the extract through the total thickness of the nail, regardless of the face in which it was applied.

The data of FTIR-ATR (Figure 7) confirmed that of PAS, as indicated by the peak of the highest concentration of ETHOSS at 1035 cm^−1^ in both nail surfaces, evaluated at 1 h and 24 h. This suggested that the extract permeated the entire thickness of the nail, regardless of the face (ventral or dorsal) on which it was applied.

## 3. Discussion

Phytochemical characterization of the ethanolic fraction of the crude extract of *S. saponaria* identified compounds with antifungal properties. The ESI-MS spectrum of the ETHOSS detected the typical saponin peaks (Figure 1), similar to those reported in a previous study [16]. For saponins peaks in the range of 650–1000 m/z, the highest intensities were seen in the peaks of 683.27, 881.50, 923.48, and 965.58 m/z. All of these, except the first one, were reported as the major peaks in a previous study. These were the bioactive compounds with antifungal properties, proving that the ETHOSS has a similar profile as the ethanolic extracts studied earlier [16].

In vitro assays proved that ETHOSS has inhibitory and fungicidal effects against dermatophyte fungi (Table 1). The three clinical isolates chosen for this study were responsible for the majority of OM cases [17]. The minimum inhibitory concentration (MIC) value of terbinafine, a FDA-approved antifungal drug to treat the dermatophyte-caused OM, was 0.125 μg/mL for all isolates [18]. In the current study, ETHOSS showed a higher MIC value than terbinafine, but it is important to highlight that this comparison may not be appropriate, as these are entirely different products. While terbinafine is a pure drug administered as a pharmaceutical formulation, ETHOSS is a crude extract, rich in many components besides saponins.

The MIC and minimum fungicidal concentration (MFC) values of ETHOSS were comparable, suggesting that it has an efficient fungicidal action, which concurs with previous findings [12,16]. In fact, ETHOSS causes changes in the fungal membrane and cell wall in the first instance of contact, causing cell lysis [19]. The MIC and MFC values ranged from 195.31 μg/mL to 781.25 μg/mL, suggesting a moderate to strong antifungal activity [20]. Importantly, the higher concentration was required for *T. rubrum*, an anthropophilic fungus known for its resistance to several antifungal drugs [13].

Previously, we showed similar action of *S. saponaria* extracts on yeasts from the *Candida* genus [19], including azole-resistant isolates [12,13]. The present findings not only corroborate the antifungal properties of ETHOSS but also reinforce its potential use in treating OM, as *Trichophyton* spp. and *Candida* spp. are the main agents responsible for the nail infections [13]. We propose that this extract may be used as a topical treatment for OM, similar to our findings with propolis extract, another natural compound [14].

At the highest value of MFC, observed for *T. rubrum*, ETHOSS induced low cytotoxicity in HeLa cells, as indicated by the small number of dead cells. This parameter allows the classification of ETHOSS as slightly cytotoxic, according to ISO 10993-5. Similar results were found for other plant extracts [19,21]. The cytotoxicity of ETHOSS was higher than that of the propolis extract [14]. Nevertheless, this level of toxicity of ETHOSS could be improved in the future by encapsulation, as was done recently for the aqueous extract of *Moringa oleifera* leaves, with potential application in wound dressing [22]. In any case, the cytotoxicity observed in the cultured cells does not pose a concern here, as we propose a possible use of ETHOSS only as a topical treatment for OM, and therefore, in its clinical use, it is not expected to affect living tissues.

The absorption profiles of this extract were characterized by PAS; the photoacoustic signal was detected in the absorption band between 300 and 650 nm (Figure 4A). Similarly, FTIR-ATR (Figure 4B) showed the presence of saponin-specific functional groups, as the absorption peak at 1660 cm^−1^ indicated the C=O bond, while those of 1514 and 1369 cm^−1^ were attributed to the C=O stretch. The 1430 cm^−1^ refers to the flexion of the C-H bond of alkanes, and the 1035 cm^−1^ band refers to the vibration of the stretched C–O bond of the alcohols and carboxylic acid present in saponins [15,23].

In the present study, ETHOSS was applied after the removal of approximately 30 μm thick layer from both the dorsal (external) and ventral (internal) nail surfaces by sanding. This is important for removing the most superficial layers of the nail and has been recommended to facilitate the diffusion of topical drugs [8].

To standardize the detection of the ETHOSS after nail permeation using PAS and FTIR-ATR, the same extract at concentrations ranging from 781.25 μg/mL to 100 mg/mL were tested in aqueous solutions. From 3125.00 μg/mL, well-defined and characteristic spectra were observed, mainly at 365 nm in PAS (Figure 6) and at 1035 cm^−1^ in FTIR-ATR (Figure 7). Thus, the detection of the components of ETHOSS by the spectroscopic tools in the human nails after its application confirmed the permeation of the extract across the nail.

Our results show that ETHOSS was able to permeate across the thickness of the nail, regardless of the face on which it was applied (Figure 6 and Figure 7). Within 1 h, the extract was able to permeate across the nail; in addition, it remained detectable in the nail till 24 h, without a second application. The ability to permeate healthy nails is crucial for a topical drug to treat OM [14,24]. In clinical settings, this property is likely to be further enhanced since fungal-infected nails are more easily penetrated by hydrophilic molecules than healthy nails [25]. To our knowledge, there is yet no commercially available plant crude extract addressing the treatment of OM. Thus, based on our observations, we propose ETHOSS as a potential option.

## 4. Methods and Materials

### 4.1. Plant Material

The dried pericarps of *Sapindus saponaria* L. fruits were collected on the campus of the Universidade Estadual de Maringá, in Maringá, Paraná, South of Brazil, a region with a humid subtropical climate, at an altitude of 551 m, latitude: 23°25′38″ South, longitude: 51°56′15″. The plant was identified by the Botany Department of UEM, and a voucher specimen (Number HUM 11710) was deposited in the UEM Herbarium, Paraná, Brazil.

### 4.2. Hydroalcoholic Extract

The hydroalcoholic extract (ETHOSS) from the pericarps of *Sapindus saponaria* L. was prepared according to Shinobu-Mesquita et al. [16]. Briefly, fruits of *S. saponaria* (400 g) were homogenized, extracted with 9:1 (*v/v*) mixture of ethanol: H_2_O, and concentrated under low pressure in a rotary evaporator at 40 °C. After the removal of the solvent, the crude extract was frozen in liquid nitrogen, lyophilized using Alpha 1-2 Martin Christ lyophilizer (Osterode am Harz, Niedersachsen, Germany), and stored frozen in a closed plastic bottle until use. For use, the extract was dissolved in ultrapure water.

Electrospray ionization mass spectrometry (ESI-MS) analysis was carried out using a model mass spectrometer (MICROMASS QUATTRO LC) (Waters Corporation Mildfort, MA, USA). The samples were diluted in methanol (chromatographic grade) and directly injected (10 μL), using nitrogen as the nebulizing and dissolving gas. The capillary tension and cone puller values were optimized for each sample. Masslynx 3.3 (Micro Mass) (Waters Corporation, Milford, MA, USA) was used for operating the equipment and data processing.

### 4.3. Fungi

We used three dermatophyte fungi, previously isolated from the patients of OM. These strains were obtained from the collection of the Medical Mycology Laboratory of the State University of Maringa, Brazil, and were deposited in the Paranaense Network of Biological Collections (TAX-online), under the following identification: *Trichophyton rubrum* CMRP 2913, *T. mentagrophytes* CMRP 2920, and *T. interdigitale* CMRP 2921. Prior to each experiment, isolates were grown on Sabouraud dextrose agar (SDA; Difco TM; Detroit, USA) for seven days at 25 °C.

### 4.4. Antifungal Activity

The antifungal properties of ETHOSS were determined in vitro by the broth microdilution method, according to CLSI (Clinical Laboratory Standards Institute, 2010, M38-A2), with some modifications for natural products [26,27]. Ten serial concentrations of ETHOSS from 195.31 to 50,000 μg/mL were prepared in RPMI 1640 (Gibco: NY, USA) with 1-glutamine (with sodium bicarbonate), 0.165 M 3-(*N*-morpholino), and propanesulfonic acid (pH 7.2) as a buffer (Sigma-Aldrich; St. Louis, USA). The fungal inoculum was adjusted to a final concentration of 5 × 10^4^ conidia/mL. The culture was incubated at 37 °C for 48 h, and the minimum inhibitory concentration (MIC) was determined by visual observation.

The minimum fungicidal concentration (MFC) was determined by plating 10 μL aliquots from the samples used in MIC determination on SDA plates. These correspond to inoculum exposed to different concentrations of ETHOSS. These plates were incubated at 25 °C for 48 h before visually assessing the growth inhibition. MFC was defined as the lowest concentration of ETHOSS that inhibited the growth of fungi in a complete, extract-free medium, according to Capoci et al. [19]. These tests were performed in duplicates. Terbinafine (0.015 to 8 μg/mL) and was used as a reference antifungal agent and positive control in the assay. We also used the reference strain *T. rubrum* ATCC 40051 as a control for reproducibility of the antifungal activity assay, according to the CLSI M38-A2 document.

### 4.5. Evaluation of Cytotoxicity of ETHOSS In Vitro Based on Cell Viability

HeLa cells were incubated at 37 °C under 5% CO2 in Dulbecco’s modified Eagle’s medium (DMEM; Sigma-Aldrich, St. Louis, MO, USA) containing 10% fetal bovine serum (FBS; Life Technologies, Itapevi, Brazil) and 1% penicillin-streptomycin (Pen Strep; Gibco, Grand Island, NY, USA). After becoming 80% confluent, the cells were detached using 25% trypsin-EDTA (Gibco, Grand Island, NY, USA), washed, and resuspended in fresh DMEM to a count of 2 × 10^5^ cells/mL. These cells were seeded in a 96-well plate and incubated for 24 h, before the addition of ETHOSS at a concentration equivalent to MFC (781.25 μg/mL). After 24 h of further incubation, the cells were stained using the Live/Dead^®^ kit under the same conditions. After incubating for 15 min, images were captured under a fluorescence microscope (EVOS FL, Life Technologies, Carlsbad, CA, USA). The cytotoxicity of the ETHOSS was inferred by the viability and morphology of cells and presented as the average of three independent experiments with three replicates.

### 4.6. Analysis of the Permeance of ETHOSS into Human Nails

#### 4.6.1. Human Nails

Fingernail fragments were collected from adult, healthy female voluntary donors and manually cut into equal-sized pieces before autoclaving at 121 °C for 20 min. The study protocol was approved by the Ethical Committee under the number 615.643/2014.

#### 4.6.2. Photoacoustic Spectroscopy (PAS)

The spectrum of the components was measured separately for each sample: nail (dorsal and ventral surfaces), ETHOSS, and commercial keratin (MP Biomedicals, Clevenlend, Ohio USA, 6358F), using the experimental setup described previously [28]. The modulated light on the surface of each sample reaches different depths, according to the modulation frequency used. The thickness of the sample that contributes to the photoacoustic signal was estimated by calculating the thermal diffusion length (μs). This parameter was mathematically defined as μs = (d/πf) 1/2, where d is the thermal diffusivity of the barrier, and f is the light modulation frequency. Using d = 10 × 10^–4^ cm^2^ s^−1^ [29], the thermal diffusivity of the nail (oμs) was 49 μm. The nail samples had an average thickness of 447 μm. The analysis was carried out in the ultraviolet and visible spectral regions, with wavelengths ranging from 300 to 650 nm, 800 W source power, and a light modulation frequency of 13 Hz.

#### 4.6.3. FTIR Coupled to Attenuated Total Reflectance (FTIR-ATR)

The structural and molecular analyses were done through FTIR-ATR, using a Fourier transformation infrared spectrometer (Vertex 70 v, Bruker Optik GmbH, Ettlingen, BW, DEU) with attenuated full reflectance accessory and diamond ATR crystal. Initially, the absorption spectra of the nail (dorsal and ventral), keratin, the main component of the nail, and ETHOSS were individually measured. The spectral range was 400 to 4000 cm^−1^ with 128 scans and a spectral resolution of 4 cm^−1^. A computer connected to the spectrometer was used to acquire data via software, with background correction and refractive index of the ATR crystal.

#### 4.6.4. Evaluation of ETHOSS on Nail by PAS and FTIR-ATR

First, the nail spectrum was analyzed by PAS and FTIR-ATR for controls without ETHOSS application. Then, using common sandpaper, an approximately 30 μm thick layer was removed from the dorsal face (external) of the nail, and 3 μL of ETHOSS were applied (Figure 3). In order to evaluate the penetration of ETHOSS into the nail, the samples were illuminated first on the dorsal side and then on the ventral side to detect the optical absorption band of ETHOSS. The same procedure was followed for the ventral face (internal) of other nail fragments. The preparations were aseptically maintained in a humid chamber at room temperature. After 1 h and 24 h, readings were taken from the reverse surface, opposite to the application of the extract. All tests were performed in triplicate using both techniques.

#### 4.6.5. Statistical Analysis

Data distributions and descriptive analysis were performed, and the average and the standard deviation (± SD) were calculated. For statistical analysis, the software RStudio version 1.0.136 (Affero General Public License) was used. Data were analyzed by one-way analysis of modification (ANOVA), and the post hoc comparisons of mediums were carried out by Tukey’s test; *p*-values < 0.05 were considered statistically significant.

## 5. Conclusions

ETHOSS showed fungicidal action on the main fungi that cause OM, low cytotoxicity, and excellent permeation into human nails. Additionally, it permeated quickly through the full-thickness of the nail (1 h), and its retention time in the nail tissue was relatively long (24 h), suggesting that a daily topical application would be effective. In addition, as ETHOSS shows high nail permeation in an aqueous solution, facilitating vehicles are not needed. Overall, ETHOSS is a plant product with great potential in treating OM either as a crude extract, or in an encapsulated form to improve its efficiency and lower the toxicity.

## Figures and Tables

**Table 1:** Evaluation of Minimum Inhibitory Concentration (MIC) and Minimum Fungicidal Concentration (MFC) of *Sapindus saponaria* L. hydroalcoholic extract (ETHOSS) against *Trichophyton* spp. isolated from patients with onychomycosis.

Fungi	MIC = MFC * of ETHOSS (μg/mL)
*T. rubrum* CMRP 2913	781.25
*T. mentagrophytes* CMRP 2920	390.63
*T. interdigitale* CMRP 2921	195.31

* MIC and MFC: correspond to the same values. CMRP (Paranaense Network of Biological Collections).
